# The drinking water contaminant dibromoacetonitrile delays G1-S transition and suppresses Chk1 activation at broken replication forks

**DOI:** 10.1038/s41598-017-13033-8

**Published:** 2017-10-06

**Authors:** Thomas Caspari, James Dyer, Nathalie Fenner, Christian Dunn, Chris Freeman

**Affiliations:** 1 Bangor University, School of Medical Sciences, Bangor, LL57 2UW United Kingdom; 2 Bangor University, Bangor Wetlands Group, School of Biological Sciences, Bangor, LL57 2UW United Kingdom

## Abstract

Chlorination of drinking water protects humans from water-born pathogens, but it also produces low concentrations of dibromoacetonitrile (DBAN), a common disinfectant by-product found in many water supply systems. DBAN is not mutagenic but causes DNA breaks and elevates sister chromatid exchange in mammalian cells. The WHO issued guidelines for DBAN after it was linked with cancer of the liver and stomach in rodents. How this haloacetonitrile promotes malignant cell transformation is unknown. Using fission yeast as a model, we report here that DBAN delays G1-S transition. DBAN does not hinder ongoing DNA replication, but specifically blocks the serine 345 phosphorylation of the DNA damage checkpoint kinase Chk1 by Rad3 (ATR) at broken replication forks. DBAN is particularly damaging for cells with defects in the lagging-strand DNA polymerase delta. This sensitivity can be explained by the dependency of pol delta mutants on Chk1 activation for survival. We conclude that DBAN targets a process or protein that acts at the start of S phase and is required for Chk1 phosphorylation. Taken together, DBAN may precipitate cancer by perturbing S phase and by blocking the Chk1-dependent response to replication fork damage.

## Introduction

Dibromoacetonitrile (DBAN) is generated at low nanomolar concentrations when bromide reacts with nitrogenous organic matter during the chlorination of drinking water^1^. A survey of 20 water supply systems in England and Wales revealed DBAN as the most abundant haloacetonitrile (HAN) (≤40.2 nM, ≤8 μg/L)^2^. Epidemiological studies in the USA linked the consumption of chlorinated water with an increased risk of bladder, brain and rectal cancer^3–5^. Very high concentrations of DBAN (>250 μM) induce cancer of the liver and stomach in rodents^6,7^. The WHO issued guidelines for DBAN (0.35 μM), dichloroacetonitrile (DCAN; 0.18 μM) and trichloroacetonitrile (TCAN; <0.007 μM) in the response to these findings^8^.

While DBAN is probably not mutagenic, it effectively alkylates DNA *in vitro* which may explain how it breaks DNA and elevates sister chromatid exchange in yeast and mammalian cells^9–11^. DBAN increases the levels of reactive oxygen species (ROS) in rat cells as indicated by a rise in 8-hydroxy-2-deoxyguanosine (8OHdG)^12,13^ and blocks aldehyde dehydrogenase^14^, dimethylnitrosamine-demethylase^15^, glutathione-S-transferase^16^, superoxide dismutase and catalase^17^ in the liver. Monobromoacetontrile (BAN) was shown to induce endoreplication in Chinese hamster ovary cells by blocking mitosis^18^. While these findings indicate a potential health risk, it is still unclear how DBAN precipitates cancer.

Using the model organism fission yeast (*Schizosaccharomyces pombe*), we report here that DBAN delays G1-S transition and specifically blocks the activation of the DNA damage checkpoint kinase Chk1 at broken DNA replication forks.

The replication machinery assembles around the DNA helicase MCM_2-7_ at dedicated chromosomal sites (origins) in a stepwise process (reviewed in^19^). The pre-replication complex forms late in G1 when DDK, a heterodimer of Cdc7/Hsk1 kinase and its activating subunit ASK/Dfp1,Dbf4, recruits Sld3 (Treslin) and Cdc45. The cell cycle regulator CDK (cyclin-dependent kinase) activates this complex subsequently at the start of S phase by loading the BRCT-domain protein Rad4 (Dbp11, TopBP1), Sld2, DNA polymerase epsilon and the GINS proteins^20^. Processive DNA replication begins when DNA polymerase alpha-primase and MCM10 associate with this structure. The lagging strand is displaced in front of the moving replication fork where Cdc45 channels it into DNA polymerase delta. The leading strand runs straight through the fork to be copied by DNA polymerase epsilon^21,22^. A drop in the nucleotide pool, which can be triggered by the RNR (ribonucleotide reductase) inhibitor hydroxyurea (HU), stalls forks in early S phase by activating the intra-S checkpoint kinase Cds1^23^. Cds1 binds to Mrc1 (Claspin) at the stalled fork when both proteins are phosphorylated by Rad3 (ATR)^24^. Mrc1 associates also with early origins during the G1-S transition, independently of the checkpoint, where it is modified by DDK^25^. The collision of replication forks with immobilised topoisomerase 1, which can be trapped on the DNA by camptothecin (CPT), triggers the phosphorylation of the DNA damage checkpoint kinase Chk1 at serine 345 by Rad3^26^. Activation of Cds1 and Chk1 both block the cell cycle activator Cdc2 (CDK1) thereby initiating a transient G2-M arrest^27^.

We report here that DBAN perturbs G1-S transition and blocks Chk1 phosphorylation at a broken replication fork without affecting the Rad3-dependent modification of other checkpoint proteins. DBAN may therefore act on a process or a protein that is required at the start of S phase and later at damaged DNA replication forks. We conclude that DBAN elicits DNA replication stress, a known driver of cancer development^28^.

## Results

### DBAN interferes with S phase

**Figure 1:**
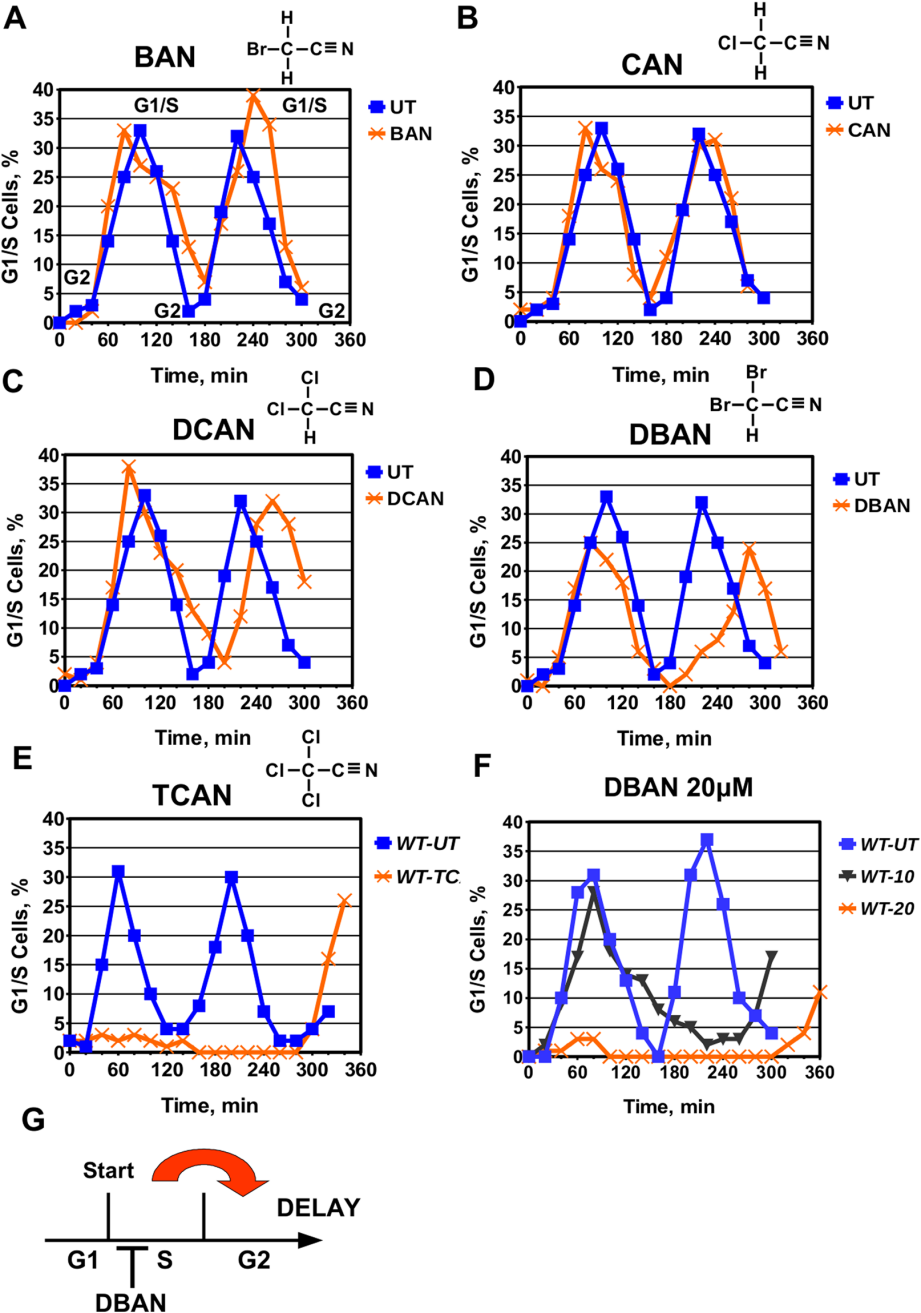
Dibromoacetonitrile (DBAN) arrests cell cycle progression in a concentration dependent manner. Wild type cells (*ade6-M210 leu1–32 ura4-D18*) were synchronised in G2 by lactose gradient centrifugation^29^ and released into rich medium (3% glucose, 0.5% yeast extract, 100 mg/L adenine) without (UT = untreated) or with 10 μM of the indicated haloacetonitriles (HANs). All HANs were diluted from a 12 mM stock solution in DMSO to a final concentration of 10 μM. (**A**) bromoacetonitrile (BAN), (**B**) chloroacetonitrile (CAN), (**C**) dichloroacetonitrile (DCAN), (**D**) dibromoacetonitrile (DBAN), (**E**) trichloroacetonitrile (TCAN), (**F**) DBAN at 10 μM or 20 μM. (**G**) DBAN affects cells in S phase which triggers a second cycle delay.

Informed by the ability of bromoacetonitrile (12 μM) to block mitosis in Chinese hamster ovary (CHO) cells^18^, we first tested whether haloacetonitriles (HANs) impact on cell cycle progression. Wild type cells were synchronised in G2 by lactose gradient centrifugation^29^ and released into medium with 10 μM bromo-, dibromo-, chloro-, dichloro- or trichloroactonitile (BAN, DBAN, CAN, DCAN, TCAN). Samples were withdrawn every 20 min to score septated G1/S cells. While the monohalogen compounds, BAN and CAN, allowed cells to complete two cell cycle rounds, their dihalogen forms, DCAN and DBAN, delayed entry into the second cycle by 40 min and 60 min, respectively (Fig. 1C,D). Unlike CHO cells^18^, DBAN-treated yeast cells arrested in G2 before the onset of mitosis (Fig. S1A,B). Since a second cycle G2 arrest is typical for drugs like hydroxyurea (HU) or camptothecin (CPT) that interfere with DNA replication, we concluded that DBAN and DCAN perturb S phase thereby triggering the G2 delay (Fig. 1G). HU stalls DNA replication forks by depleting the dNTP pool, whereas CPT breaks forks by immobilising topoisomerase 1 in front of the advancing replication complex. Both events delay onset of mitosis through the Rad3-dependent activation of the checkpoint kinases Cds1 and Chk1, respectively^27,30^. To investigate whether the DBAN-induced S phase perturbations activate the checkpoint, we synchronised a checkpoint-defective *Δrad3 Δtel1* double mutant (Tel1/ATM is the second checkpoint kinase besides Rad3/ATR) in G2 and released cells into medium with or without 10 μM DBAN. Interestingly, the checkpoint deficient strain arrested like wild type for 80 min (Fig. S1A,C) showing that the G2 delay is independent of the DNA damage response. An unexpected observation was made when we analysed the cell cycle impact of trichloroacetonitrile. Unlike DBAN or DCAN, TCAN (10 μM) blocked entry into the first cycle for around 280 min (Fig. 1E). Since such a first cycle arrest is typical for agents that break DNA^30^ and because HANs were linked with DNA breaks in mammalian cells^10^, we tested whether they increase the phosphorylation of histone 2AX at S129 by Rad3 and Tel1, an established marker of chromosomal breaks^31^. Intriguingly, all HANs (BAN, CAN, DBAN, TCAN), with the exception of DCAN, reduced the phosphorylation of H2AX showing that DNA breaks are not the cause of the arrest (Fig. S1D). Since H2AX is also phosphorylated during unperturbed S phase^31^, this drop may be caused by depleting the pool of S phase cells due to the G2 arrest. We can however not exclude the possibility that the HANs affect H2AX phosphorylation more directly since BAN and CAN do not show a strong cell cycle arrest but reduce H2AX modification (Fig. 1A,B; Fig. S1D). The decline in H2AX phosphorylation was concentration dependent starting at 8 μM DBAN (Fig. S1E). We next tested whether the first cycle arrest is unique to TCAN and found that a higher concentration of DBAN (20 μM) also delayed cells in the first G2 phase (Fig. 1F). This implies that TCAN is more effective than DBAN in eliciting this response. As in the case of the second cycle arrest, the DNA damage checkpoint was not required (Fig. S1F). A checkpoint-defective *Δcds1 Δchk1* double mutant delayed mitosis for 220 min, although this arrest was 40 min shorter compared to wild type cells (Fig. S1F). We did not further investigate this first cycle arrest as we wanted to learn more about how DBAN perturbs S phase given the importance of DNA replication stress in malignant transformation^28^.

### DBAN delays G1-S progression

**Figure 2:**
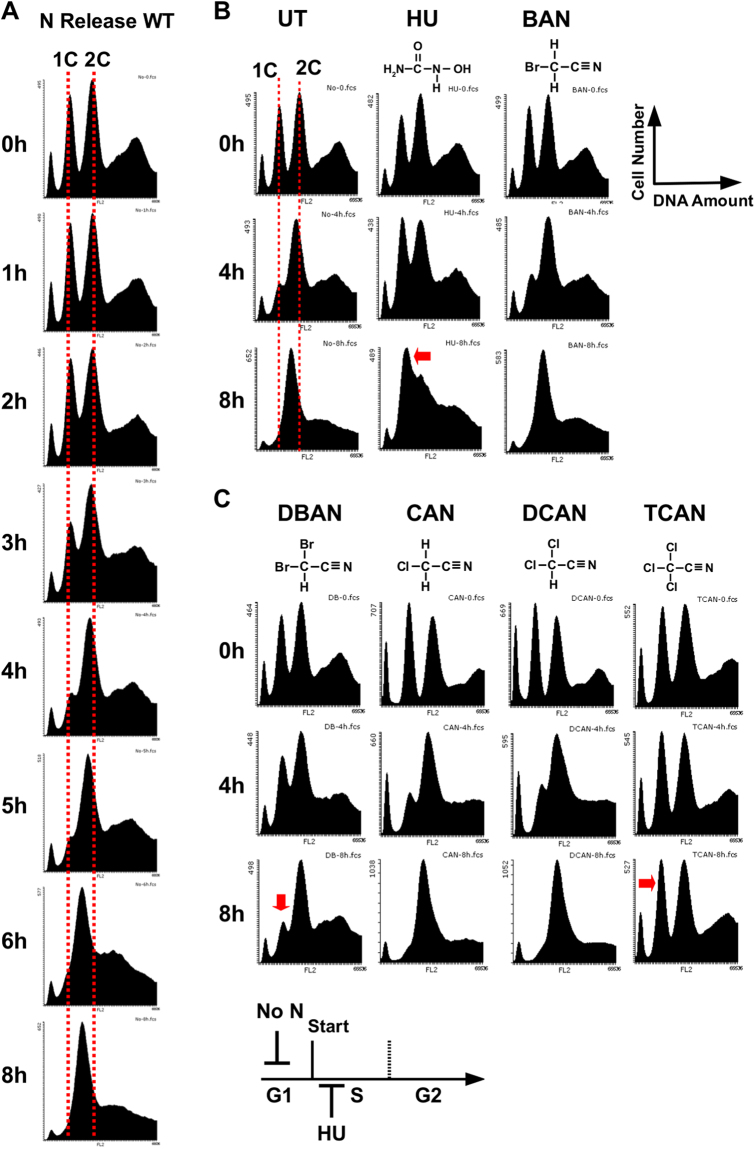
DBAN and TCAN delay G1-S transition. (**A**) Wild type cells without auxotrophic markers were synchronised in G1 by nitrogen starvation in minimal medium (3% glucose, 0.67% nitrogen base w/o amino acids and ammonium sulphate) at 30 °C^29^. Cells were washed and released at T = 0 h into pre-warmed minimal medium with ammonium sulphate. Samples were withdrawn at the indicated time points and the DNA content was measured by flow cytometry^29^. The dotted lines indicate 1 copy of the chromosomes (1 C, G1) and two copies (2 C, G2), respectively. (**B**,**C**) DNA content at the start of the experiment (0 h), at 4 h and 8 h post-release. Untreated cells (UT) progress from G1 (1 C) to G2 (2 C) within 4 h. This progression is slowed down by DBAN but blocked by TCAN (indicated by the arrows). The final concentration of all HANs is 10 μM. In a control experiment, cells were arrested in early S (1 C) with 15 mM hydroxyurea (HU).

To map the execution point of DBAN in S phase, wild type cells were enriched in G1 by nitrogen starvation and released back into the cell cycle by replenishing the medium with a nitrogen source^29^. The DNA content was measured by flow cytometry over 8 hours to monitor progression from G1 (1 copy of the chromosomes, 1 C) into G2 (two copies, 2 C) (Fig. 2). While untreated cells reached G2 4 h post-release (Fig. 2A), DBAN (10 μM) delayed exit from G1 whereas TCAN (10 μM) blocked cells in G1 (Fig. 2B,C). We also arrested cells in early S phase with 15 mM HU to have an internal marker for unreplicated DNA (Fig. 2B). Eight hours after the release from G1, only HU and TCAN treated cells remained arrested with a 1 C DNA content, while DBAN permitted the completion of DNA replication (Fig. 2B,C). This shows that DBAN only delays G1-S transition, whereas TCAN blocks this step more effectively. None of the other HANs (BAN, CAN, DCAN) had a similar effect on G1-S transition (Fig. 2B,C).

To exclude the possibility that the G1 arrest protocol impacts on this interesting finding, we synchronised *nda3-KM311* cells in mitotic prophase. This cold sensitive beta-tubulin mutant stops with condensed chromosomes without a mitotic spindle at 20 °C and returns to the cell cycle within minutes upon a temperature up-shift to 30 °C^32^. In line with the first experiment, TCAN prevented the accumulation of G2 cells whereas DBAN only delayed it (Fig. S1G).

We next arrested cells in early S phase by incubating a wild type strain in 15 mM HU for 3.5 h^29^ to test whether addition of DBAN or TCAN (10 μM) after the G1-S transition would still delay the cell cycle. While the latter was not the case for DBAN (Fig. S2B), TCAN-treated cells delayed progression through S phase by 20 min compared to untreated cells (Fig. S2C). Since DNA replication was complete within 60 min in untreated cells (Fig. S2A), a 20 min difference (1/3 of S phase) may well be significant.

Taken together, these data show that DBAN delays G1-S transition while allowing cells to complete DNA replication. In contrast, TCAN blocks cells effectively before the G1-S transition and slightly delays DNA replication. This conclusion is in line with the higher potency of TCAN as a G2 blocker (Fig. 1E).

### DBAN affects DNA polymerase delta

**Figure 3:**
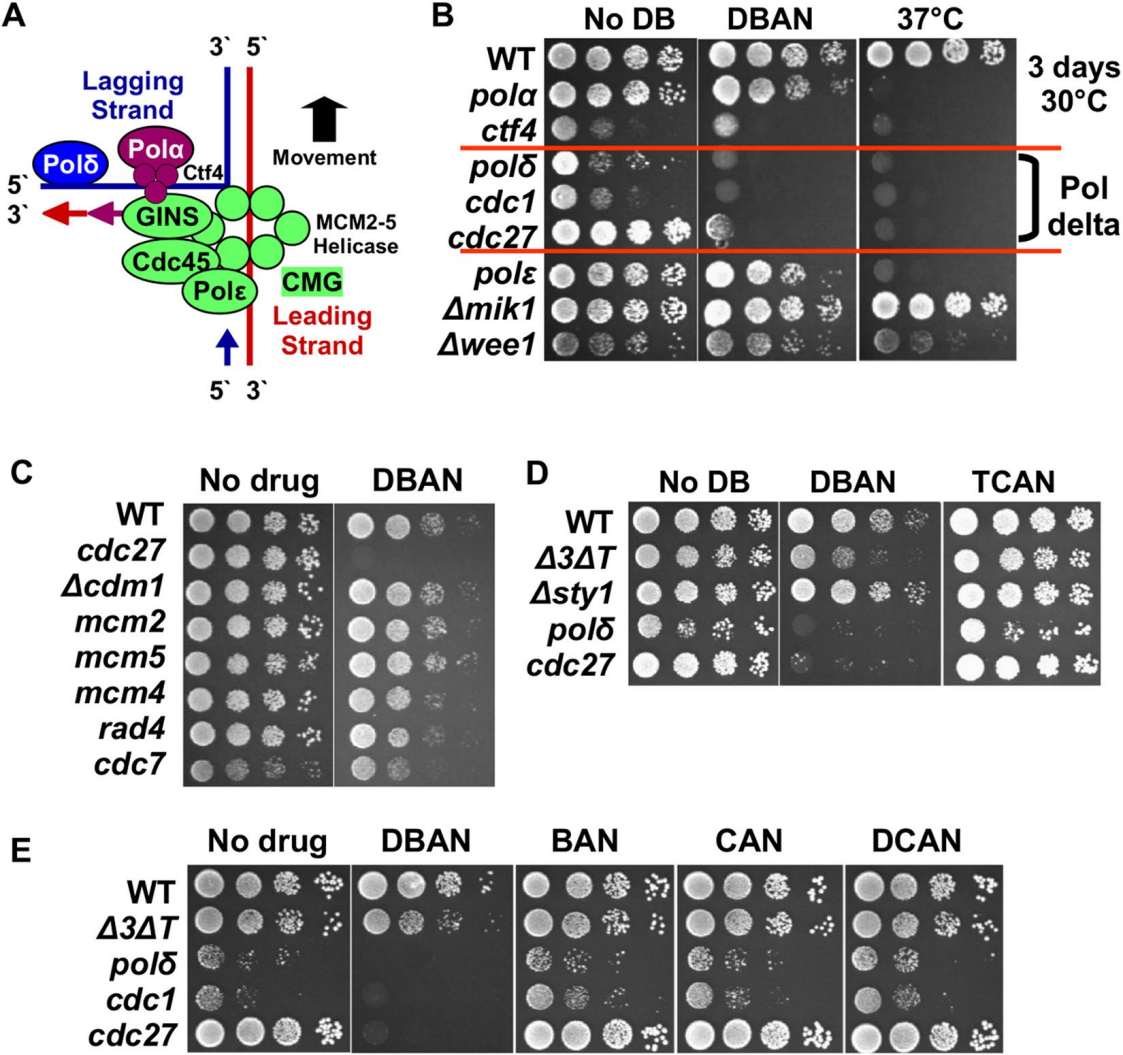
DBAN kills cells mutated in DNA polymerase delta. (**A**) Model of the replication fork (adapted from^22^). (**B**–**E**) Serial dilutions of the indicated strains were applied to rich medium plates at 30 °C. One plate was incubated at 37 °C. Incubation time: 3 days. The final concentration of DBAN was 10 μM, but 20 μM for TCAN. The mutant alleles are: pol alpha (*swi7.H4*), Ctf4 (*mcl1-1*), pol delta (*cdc6.23*, *cdc27.P11*, *cdc1.p13*), pol epsilon (*cdc20.M10*), S phase cell cycle inhibitor Mik1 (*mik1::ura4*+ ), cell cycle inhibitor Wee1 (*wee1::ura4*+ ), *Δcdm1* (non-essential pol delta subunit, *cdm1::ura4*+ ), MCM2 (*cdc19.P1*), MCM4 (*cdc21-M68*), MCM5 (*nda4-108*), DDK/Cdc7 (*hsk1-1312*), Rad4 (TopBP1) (*rad4.116*), MAP kinase Sty1 (*sty1::ura4*+ ). Final concentration of BAN, CAN and DCAN is 10 μM.

Since DBAN allows DNA replication to proceed after an initial G1-S delay (Figs 2C, S1G), we tested whether this affects the three replicative DNA polymerases, alpha (Pol1, *swi7*), epsilon (Pol2, *cdc20*) or delta (Pol3, *cdc* *6*). Since these essential genes cannot be deleted, we used temperature-sensitive mutants at the semi-restrictive temperature of 30 °C. Serial dilutions of the strains were applied to rich medium plates containing no HAN or 10 μM DBAN. We also incubated one plate at 37 °C to confirm the temperature sensitivity. While mutations in the three essential subunits of Pol delta (*cdc6*.*23* [catalytic]*, cdc* *2* *7.P11* [non-catalytic], *cdc1.P13* [non-catalytic]) impaired cell viability, mutations in the catalytic subunits of Pol epsilon (*cdc20.M10*) or Pol alpha (*swi7.H4*) did not (Fig. 3B). Interestingly, deletion of the fourth, non-essential Pol delta subunit, *cdm1*, had also no effect (Fig. 3C). We next tested mutations in the MCM_2-7_ helicase that unwinds the DNA template (MCM2 [*cdc19.P1*], MCM4 [*cdc21-M* *6* *8*] or MCM5 [*nda4-108*]), but failed to detect loss of viability (Fig. 3C). Also no impact on cell growth was found for mutations in DDK (Cdc7/Hsk1) kinase (*hsk1-1312*) and in the replication factor Rad4 (TopBP1) (*rad4.11* *6*) (Fig. 3C). Whether a mutation in Ctf4 (*mcl1-1*), which binds Pol alpha to the replication complex, impairs cell viability was difficult to judge since the strain grew very poorly even in the absence of DBAN (Fig. 3B). Interestingly, none of the DNA pol delta mutants showed a growth defect on TCAN plates even at 20 μM (Fig. 3D). This was unexpected given the high impact of TCAN on cell cycle progression. A possible explanation is provided by the replication delay caused by TCAN (Fig. S2C) that may prevent loss of viability of pol delta mutants. The latter mutants were also not sensitive to CAN, BAN or DCAN at 10 μM (Fig. 3E).

### DBAN overcomes the intra-S arrest of a pol delta mutant

**Figure 4:**
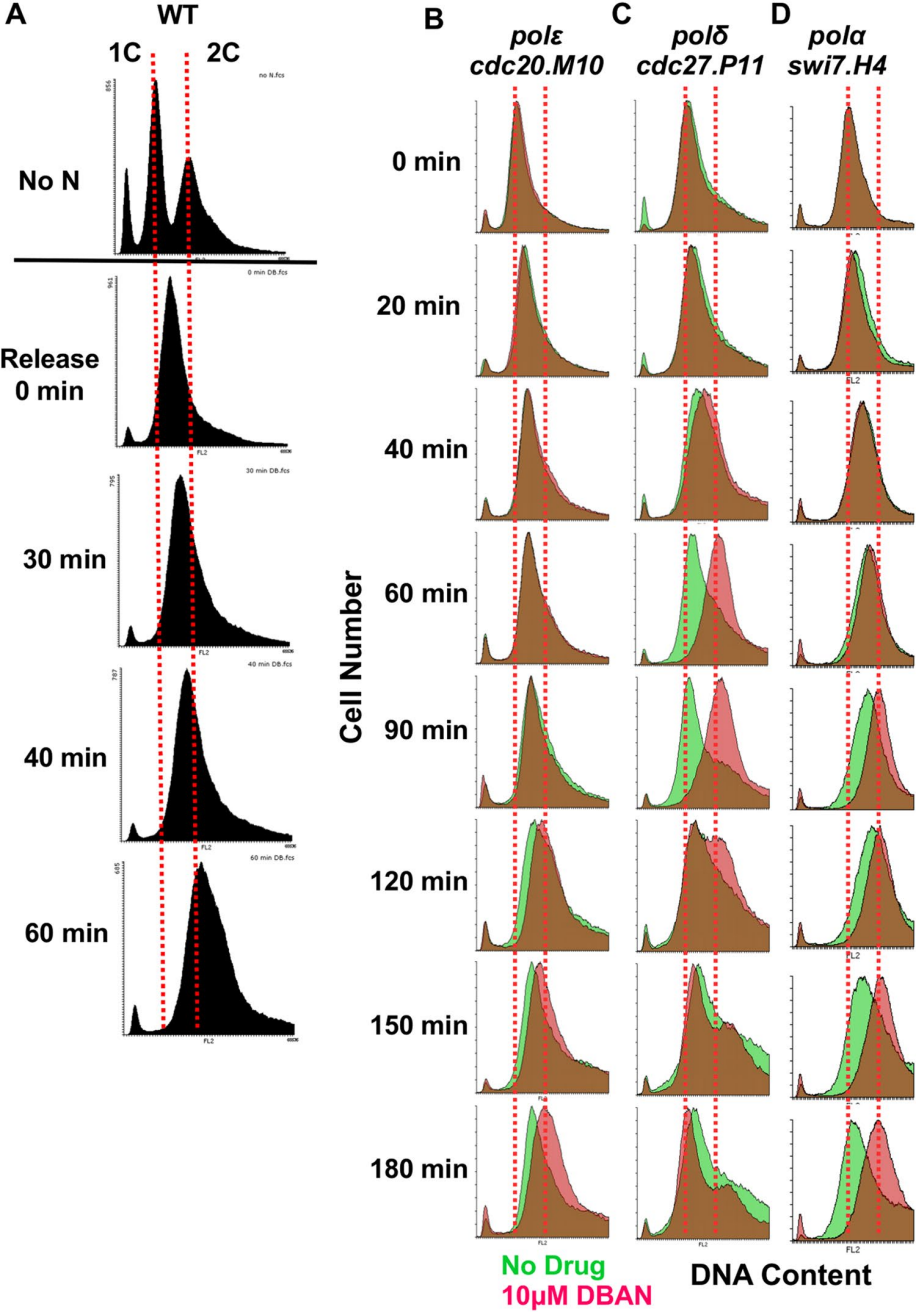
DBAN advances DNA replication in a pol delta mutant. (**A**) Wild type cells (*ade6-M210 leu1–32 ura4-D18*) were grown in rich medium at 30 °C and synchronised in early S phase for 3.5 h in 15 mM hydroxyurea (HU)^29^. HU was washed out and cells were released into pre-warmed rich medium. The DNA content was measured at the indicated times. The dotted lines indicate 1 copy of the chromosomes (1 C, G1) and two copies (2 C, G2), respectively. The DNA content of nitrogen starved cells (no N) was measured in a parallel experiment to have an internal standard. (**B**–**D**). The indicated mutant strains were HU-synchronised and released into rich medium at 30 °C with or without 10 μM DBAN. The green histogram is the DNA content of untreated cells, the red histogram is the DNA content in the presence of DBAN. The brown colour indicates that both histograms overlap.

To find out why pol delta mutants are DBAN sensitive, we synchronised wild type, pol alpha (*swi7.H4*), pol delta (*cdc* *2* *7.P11*) and pol epsilon (*cdc20.M10*) strains in early S phase using the HU arrest protocol^29^. After HU was washed out, cells were released into medium with or without 10 μM DBAN. Flow cytometry showed that untreated wild type cells completed S phase within 60 min (Fig. 4A). As previously reported^33^, the untreated mutant strains delayed S phase progression at the semi-permissive temperature of 30 °C (Fig. 4B–D). While DBAN had no impact on S phase within the first 60 min in the case of the pol alpha and pol epsilon mutants, it did significantly advance DNA replication of the pol delta (*cdc27.P11*) strain (Fig. 4C). This advancement was clearly detectable at the 60 min and 90 min time points. After 2 h, the DBAN-treated *cdc27.P11* cells had initiated already the next cell cycle round compared to the untreated sample (Fig. 4C). Cdc27 connects the catalytic (Cdc6) and non-catalytic (Cdc1) subunits, and binds Pol delta to the DNA sliding clamp PCNA^34^. DBAN also advanced DNA replication in the other two mutant strains but later and to a lesser extent (Fig. 4B,D).

These results imply that DBAN abolishes the intra-S phase arrest of the pol delta (*cdc27.P11*) strain, which may be linked with its loss of viability on DBAN plates (Fig. 3B). Since the viability of pol delta mutants depends on Chk1 kinase^33,35^ we next tested whether DBAN interferes with the activation of the checkpoint kinases Cds1 and Chk1.

### DBAN suppresses the activation of Chk1

**Figure 5:**
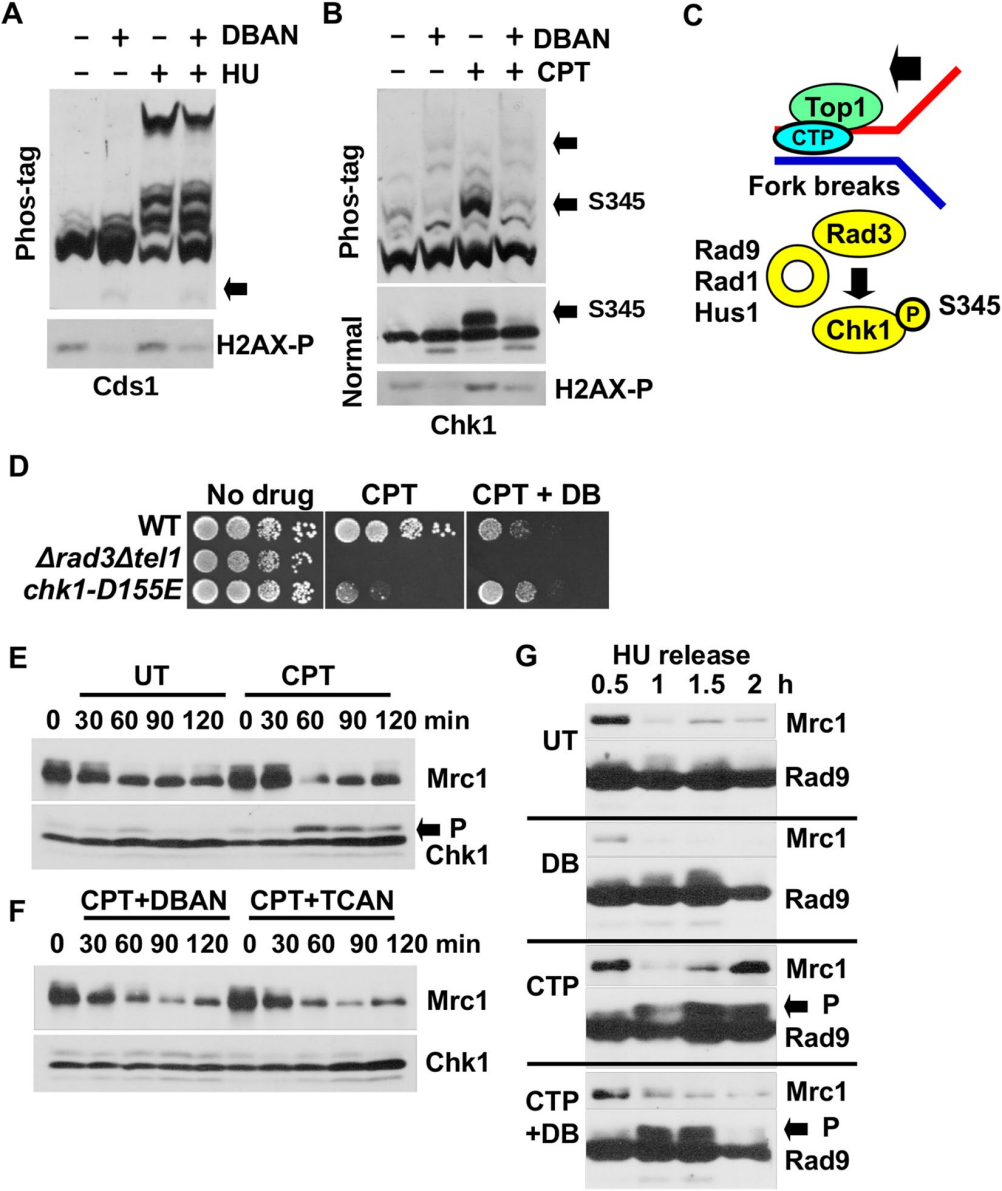
DBAN suppresses Chk1 phosphorylation. (**A**,**B**) *cds1-His*_*6*_ *HA*_*2*_ (55 kDa) and c*hk1-HA*_*3*_ (60 kDa) cells were grown in rich medium at 30 °C and treated for 4 h with 10 μM DBAN, 12 μM camptothecin (CPT), 12 mM hydroxyurea (HU) or the combination as indicated. Total protein extracts were loaded on a 6% phostag gel. Full phostag gels are shown. H2AX-S129-P was detected on a 20% acrylamide (37.5:1 acrylamide:bisacrylamide) gel. The Chk1 shift was detected on a 10% (100:1 acrylamide:bisacrylamide) gel. H2AX and Chk1 (normal) panels were cropped. The full images are shown in Figs S3 and S4, respectively. The arrows indicate the smaller Cds1 band, Chk1-S345 phosphorylation and the hyper-phosphorylated forms of Chk1. (**C**) Model of Chk1 activation by Rad3 at broken replication forks. CPT immobilises Topoisomerase 1 and the Rad9-Rad1-Hus1 ring aids Chk1 phosphorylation. Rad9 is also phosphorylated by Rad3^41^. (**D**) Drop test of the indicated strains. Chk1-D155E-HA_3_ is a kinase-dead mutant. DBAN: 10 μM; CPT: 12 μM. (**E**,**F**) c*hk1-HA*_*3*_ cells were HU synchronised (3.5 h, 15 mM HU, rich medium) and released into medium without (UT) or with CPT (12 μM), CPT (12 μM) + DBAN (10 μM) or CPT (12 μM) + TCAN (10 μM). Total protein extracts were separated on a 8% (Mrc1) and 10% (Chk1) acrylamide gel. P = Phospho-Chk1-S345 shift band). (**G**) rad9*-HA*_*3*_ cells were HU-synchronised and released into rich medium without (UT) or with CPT (12 μM), DBAN (10 μM) or CPT (12 μM) + DBAN (10 μM). P = Phospho-Rad9 shift band. Full images: Mrc1: Fig. S5; Chk1: Fig. S6; Rad9: Fig. S7.

To find out whether DBAN perturbs activation of the intra-S checkpoint kinase Cds1 at stalled forks, asynchronous *cds1-His*_*6*_ *HA*_*2*_ cells^36^ were incubated with 10 μM DBAN, 12 mM HU or with both chemicals simultaneously for 4 h. Total protein extracts were loaded onto a 6% phos-tag SDS gel to assay the phosphorylation status of Cds1. Phostag electrophoresis reveals the phosphorylation pattern of proteins as their mobility is inversely related to the extent of their modification^37^. While DBAN did not promote the modification of Cds1, the kinase was intensively phosphorylated when DNA replication forks stalled in the presence of HU. Although DBAN did not impact on this hyperphosphorylation, it induced a faster migrating band (Fig. 5A). The latter band could be a hypophosphorylated form of Cds1.

We then repeated this experiment with a *chk1-HA*_3_ strain^38^ but replaced HU with 12 μM camptothecin (CPT) to break DNA replication forks. In contrast to Cds1, DBAN effectively suppressed Chk1 phosphorylation at serine 345 (Fig. 5B). On normal SDS page, S345 phosphorylation was detected as a band shift, as previously reported, which disappeared upon DBAN exposure (Fig. 5B)^38^. DBAN also induced very slowly migrating, hyperphosphorylated bands of Chk1 independently of CPT. The suppression of Chk1 phosphorylation at damaged replication forks could explain why DBAN impairs the viability of DNA polymerase delta mutants which rely on this kinase for survival (Fig. 3B)^35^.

To test whether DBAN is an inhibitor of Rad3 kinase, we replaced CPT with MMS (methyl-methanesulfonate) that damages DNA by alkylation^39^. Since DBAN did not to block the MMS-induced phosphorylation of Chk1 (Fig. S1H), it is unlikely that the HAN impairs Rad3 kinase directly. We then exposed wild type cells (*chk1-HA*_3_) to CPT or to the combination of DBAN and CPT on plates to gauge whether replication forks still break. The latter seems to be the case as DBAN rendered wild type cells (*chk1-HA*_3_) CPT sensitive (Fig. 5D). This increase in sensitivity, which was similar to the sensitivity of a kinase-dead *chk1* mutant (*chk1-D155E-HA*_3_), indicates that forks still break while DBAN prevents activation of Chk1. We can however not exclude the possibility that CPT generates a block to DNA replication like a mutation in DNA polymerase delta that renders cells sensitive to DBAN.

To find out when DBAN acts on Chk1 in the cell cycle, we synchronised *chk1-HA*_3_ cells in early S phase with 15 mM HU and released them into medium with CPT (12 μM) or with CPT and DBAN or TCAN (10 μM). In line with the idea that Chk1 is activated once bulk DNA synthesis had been completed^40^, the shift band of Chk1, indicative of serine 345 phosphorylation, appeared 60 min post-release after the levels of the DNA replication marker Mrc1 had declined (Fig. 5E)^24^. Since Chk1 is weakly phosphorylated during the HU arrest, all samples displayed a weak shift band that increased strongly when forks were damaged by CPT (Fig. 5E, bottom panel). The presence of DBAN or TCAN effectively suppressed activation of Chk1 (Fig. 5F). Since Rad3 modifies also the Rad9 subunit of the 9-1-1 checkpoint ring at broken forks (Fig. 5C)^41^, we repeated this experiment with a HU-synchronised *rad9-HA*_3_ strain^42^. As in the case of Chk1, Rad9 phosphorylation is detectable as a band shift. This shift was not affected by DBAN strongly suggesting that Chk1 is specifically targeted by the haloacetonitrile (Fig. 5G). We noticed however that the Rad9 phosphorylation peaked 30 min earlier in the presence of DBAN (Fig. 5G, panels 3 + 4). The phos-tag assay did not reveal any changes in the Rad9 phosphorylation pattern which were DBAN specific (Fig. S1I).

Collectively, these results suggest that DBAN kills pol delta mutants (Fig. 3B) and renders wild type cells sensitive to camptothecin (Fig. 5D) by preventing the phosphorylation of Chk1 at damaged DNA replication forks.

## Discussion

**Figure 6:**
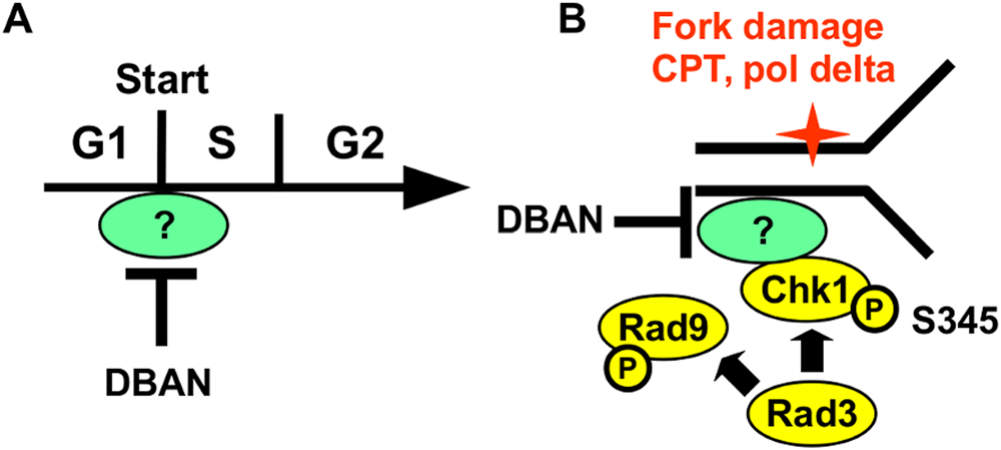
(**A**) DBAN blocks G1-S transition. (**B**) DBAN prevents Chk1 phosphorylation by Rad3 at a broken fork. The details of the model are discussed in the main text.

The evidence presented here reveals novel activities of DBAN at two stages during the cell cycle, at the G1-S transition and later at damaged replication forks (Fig. 6). DBAN affects both processes in a negative way as it delays entry into S phase (Fig. 2) and suppresses phosphorylation of Chk1 (Fig. 5B,F) without affecting the activation of Rad9 (Fig. 5G) or Cds1 (Fig. 5A). Replication fork damage can originate from the inhibition of topoisomerase 1 by camptothecin or from mutations in the lagging strand DNA polymerase delta^26,33^. Collectively, these findings imply that DBAN blocks an event or protein that is required for both entry into S phase and activation of Chk1 at damaged forks (Fig. 6). Early in S phase, human and yeast cells phosphorylate the histone H2AX in a cell cycle specific manner independently of DNA breaks^31,43^. The same chromatin modification is later required for the recruitment of Crb2 (53BP1) to a broken fork where the scaffold protein associates with Chk1^44,45^. DBAN and TCAN may therefore compromise both processes by reducing H2AX phosphorylation (Fig. S1D,E). For example, the HANs may up-regulate a phosphatase, like human PP2A-B56ϵ, that dephosphorylates H2AX at damaged forks upon CPT treatment^46^. What argues against this model is the survival of the DNA pol delta mutants in the presence of BAN, CAN or TCAN (Fig. 3) which all reduce H2AX phosphorylation (Fig. S1D). An alternative explanation is provided by the dual function of Rad4/Cut5 (TopBP1) during G1-S transition and in the activation of Chk1^47,48^. Rad4 associates with Sld3 and Sld2/Drc1 at start and with Crb2 at broken DNA^45,47^. What argues however against Rad4 is its requirement for the activation of Cds1 at stalled forks^48^ which is not affected by DBAN (Fig. 5A). A third candidate is DDK kinase as it initiates the assembly of the replication complex at the end of G1 (reviewed in^19^) and terminates Chk1 activation by phosphorylating the Rad9 subunit of the 9-1-1 complex^49^. We have however not found any evidence that the Rad9 phosphorylation pattern changes upon DBAN exposure (Fig. S1I). A fourth possibility is provided by Mrc1 (Claspin) that associates with early replication origins at start^25^ and recruits human Chk1 to broken forks^50^. A similar interaction between *S. pombe* Chk1 and Mrc1 has not yet been reported. Finally, DBAN may act directly on Chk1 as indicated by its hyper-phosphorylation (Fig. 5B). Chk1 is required in *S. pombe* and human cells at the start of the G1-S transition^51,52^ and at broken forks^26^. Further work will however be required to dissect the different possibilities that are of great interest as it is still enigmatic why human Chk1 occupies a dominant role in S phase whereas yeast Chk1 appears to act mainly in G2 (Fig. 5E)^40^.

DBAN is known to block a large number of enzymes *in vitro* ^7^ but it is as yet unclear how it interacts with proteins. Halogen atoms like bromine and chlorine have a positive charge, known as the alpha-hole, that can make electrostatic contacts with the protein backbone or amino acid side chains^53^. While bromine interacts preferentially with the side chain of arginine, chlorine prefers leucine^54^. Whether this explains why TCAN is a stronger cell cycle inhibitor than DBAN (Fig. 1) is difficult to tell as it is unclear to which protein they bind. It is even not entirely safe to conclude that they bind to the same protein. DBAN and TCAN behave in a similar way regarding the G1-S delay (Fig. 2) and the inhibition of Chk1 phosphorylation (Fig. 5F). Both HANs differ however in their lethality when DNA polymerase delta is mutated (Fig. 3D). Although this appears to contradict the earlier notion that DBAN kills these mutant strains by blocking Chk1 phosphorylation at damaged forks, it could be explained by the ability of TCAN to delay DNA replication (Fig. 4C). If this delay were to prevent fork damage in pol delta mutant cells, the inhibition of Chk1 would not affect cell viability.

Although DNA replication stress is a good explanation for why DBAN (10 μM) triggers a second cycle delay (Fig. 1D), it would not provide an answer to why 20 μM DBAN block cells in the first G2 (Fig. 1F). The concentration dependency implies that DBAN has either more than one target in cells with different affinities for the HAN or that DBAN upregulates the expression or activity of a protein in a concentration dependent manner. From the different options discussed earlier, the up-regulation of a phosphatase like PPA2 would connect the diverse cell cycle activities of DBAN and TCAN. Interestingly, induction of PPA2 arrests cells in G2 independently of the DNA damage checkpoint when the accessory protein Vpr of human immunodeficiency virus type 1 (HIV-1) is over-expressed in *S. pombe* ^55^. Since this arrest shares a similar independence from the Rad3-Tel1 checkpoint as the G2 arrest produced by DBAN and TCAN (Figs 1E,F; S1F), it is quite possible that both HANs delay cell cycle progression in G2 through a mechanism that involves the activation of a phosphatase acting on the cell cycle machinery.

The final point to consider is whether DBAN, which is frequently found in water supply systems^2^, posses a serious cancer risk. The concentration of 10 μM used in this study is approximately 30 times higher than the WHO guideline of 0.35 μM (70 μg/L). It is therefore unlikely that DBAN levels reach such high concentrations in tap water. The peak concentration found in water supply systems in Western Australia, for example, was 0.13 μM (26.6 μg/L)^56^, whereas the peak value in the United Kingdom was with 0.04 μM (8 μg/L) much lower^2^. It is however not yet clear whether haloacetonitriles accumulate over a longer consumption period in the liver, gastrointestinal tract or the kidneys^7^. The latter may explain why the consumption of chlorinated drinking water was linked with cancer^3^.

## Materials and Methods

### Yeast Strains

The genotype of the strains used in this study is *ade6-M210 leu1–*3*2 ura4-D18*. Wild type cells contained no additional mutation and the different mutant alleles are mentioned in the text. The *cds1-HA*_*2*_ *His*_*6*_ *[URA4 + ] ura4-D18* strain is described in^36^ and the *chk1-HA*_3_ strain in^38^. Before cells were synchronised in G1 by nitrogen starvation, all auxotrophic markers were crossed out.

### Cell synchronisation

Cells were synchronised as described in^29^. HU was used at a final concentration of 15 mM for 3.5 h at 30 °C in rich medium. For the G1 arrest, cells without auxotrophic markers were first grown in minimal medium with nitrogen before being transferred to minimal medium without nitrogen for 16 h at 30 °C. Lactose gradients were centrifuged for 8 min at 800 rpm. The *nda* *3* *-KM311* mitotic arrest was performed in rich medium as reported in^57^.

### Flow cytometry

The DNA content was measured using a CUBE 8 (Sysmex) instrument as described in^29^. The histograms were produced using the free Flowing Software (http://flowingsoftware.btk.fi/).

### Phos-tag SDS page

Phostag gels (6%) were prepared and run as reported in^58^.

### Antibodies

Anti-HA antibody (BioScource, Covance MMS-101P-200), anti-Mrc1 antibody (ABCAM, ab188269), anti-Cdc2 antibody (ab5467) and anti-Histone 2AX-S129-P antibody (ab17576). Secondary mouse-HRP (Dako, P0447), secondary rabbit-HRP (Dako, P0217).

### Protein extracts

Total protein extracts were prepared as described in^42^.

### Chemicals

Bromoacetonitrile (Sigma Aldrich, 242489), dibromoacetonitrile (Alfa Aesar, A16994), chloroacetonitrile (Sigma Aldrich, C19651), dichloroacetonitrile (Alfa Aesar, A10612), trichloroacetonitrile (Sigma Aldrich, T53805), phostag-acrylamide (AlphaLabs, AAL-107, 300-93523), hydroxyurea (Formedium, HDU0025).

## Electronic supplementary material

Supplementary Information
