# Supplementary material for: The drinking water contaminant dibromoacetonitrile delays G1-S transition and suppresses Chk1 activation at broken replication forks
Source: Sci Rep. 2017 Oct 6;7:12730. doi: 10.1038/s41598-017-13033-8 (PMC5630572; doi:10.1038/s41598-017-13033-8)
Supplement: Supplementary file 1 — Supplementary Information [file 41598_2017_13033_MOESM1_ESM.pdf]

Supplementary Materials for

The drinking water contaminant dibromoacetonitrile delays G1-S transition and suppresses Chk1 activation at broken replication forks

Thomas Caspari[1]*, James Dyer[1,2], Nathalie Fenner[2], Christian Dunn[2] and Chris Freeman[2]

1: Bangor University, School of Medical Sciences, Bangor LL57 2UW, United Kingdom

2: Bangor University, Bangor Wetlands Group, School of Biological Sciences

* Corresponding Author: Dr Thomas Caspari, Bangor University, School of Medical Sciences, Bangor LL57 2UW, United Kingdom, email: t.caspari@bangor.ac.uk; phone 0044-(0)-1248382526

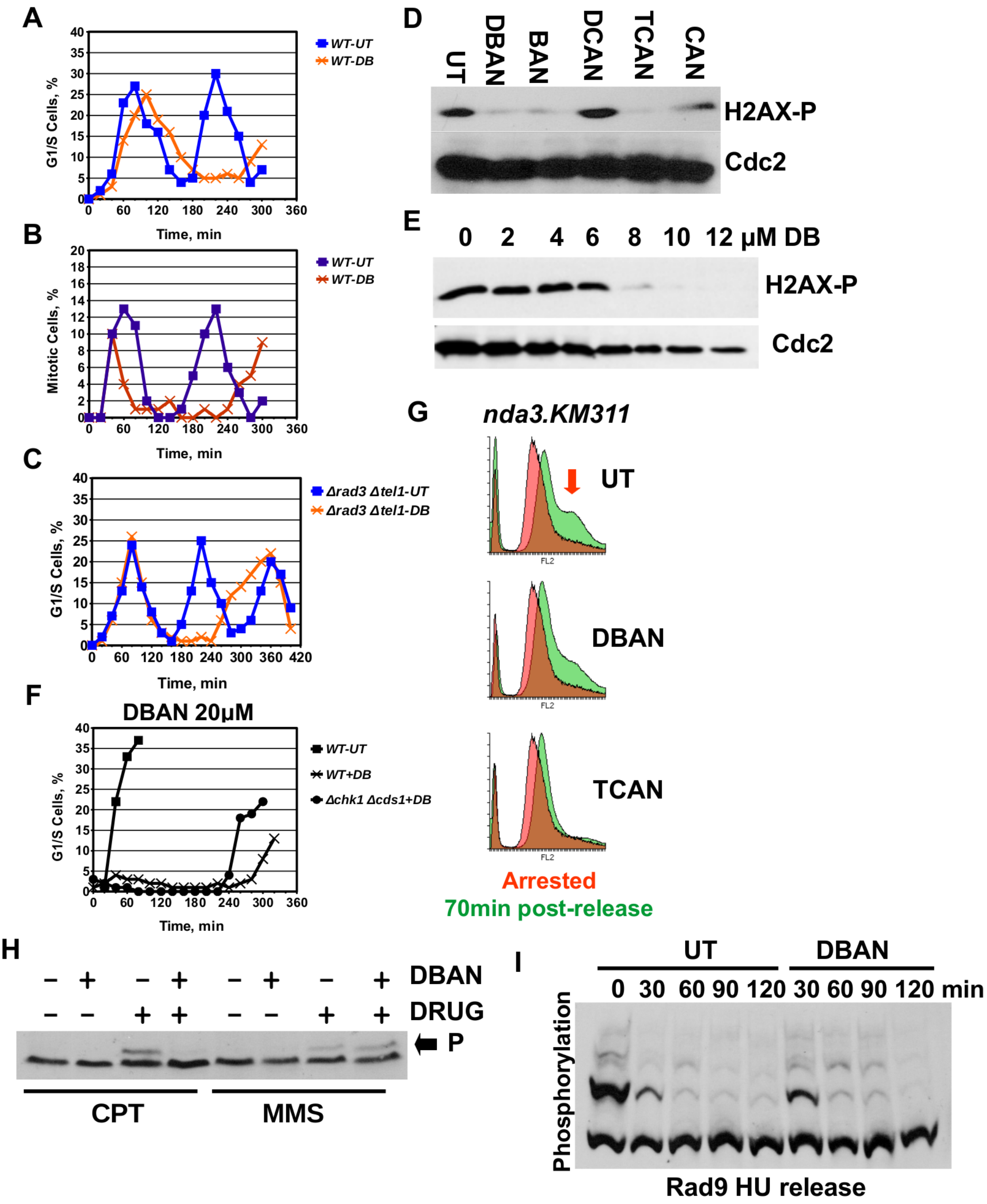

Supplementary Figure S1.

(A-B) Wild type cells (*ade6-M210 leu1–32 ura4-D18*) were synchronised by lactose gradient centrifugation in G2 and released into rich medium without (UT) or with 10μM DBAN (DB). (a) septated G1/S cells, (b) mitotic cells. **(C)** Checkpoint defective

rad3::ade6+ tel1::leu2+ cells. **(D)** Wild type cells were treated with 10μM of the indicated haloacetonitriles for 3h at 30°C. Phosphorylated H2AX-S129 was detected using a phosphospecific antibody. Cdc2 = loading control. For specificity of the anti-H2AX-S129-P antibody see Figure S3. **(E)** Concentration dependency of the decline in H2AX phosphorylation. **(F)** The indicated strains were G2-synchronised and exposed to 20μM DBAN. **(G)** *nda3-KM311* mutant cells were synchronised at 20°C for 8h and released into rich medium at 30°C. The DNA content of the arrested cells (red) and of cells 70 min post-release (green) is shown. Brown indicates that both hisograms overlap. The arrow highlights replicating cells. **(H)** *chk1-HA*$_3$ cells were treated with 0.05% MMS (methyl-methanesulfonate) or 12μM CPT with our without 10μM DBAN for 3h. For specificity of the anti-HA antibody see Figure S4. **(I)** Phos-tag analysis of the Rad9 protein samples shown in Figure 5G. The full image is shown.

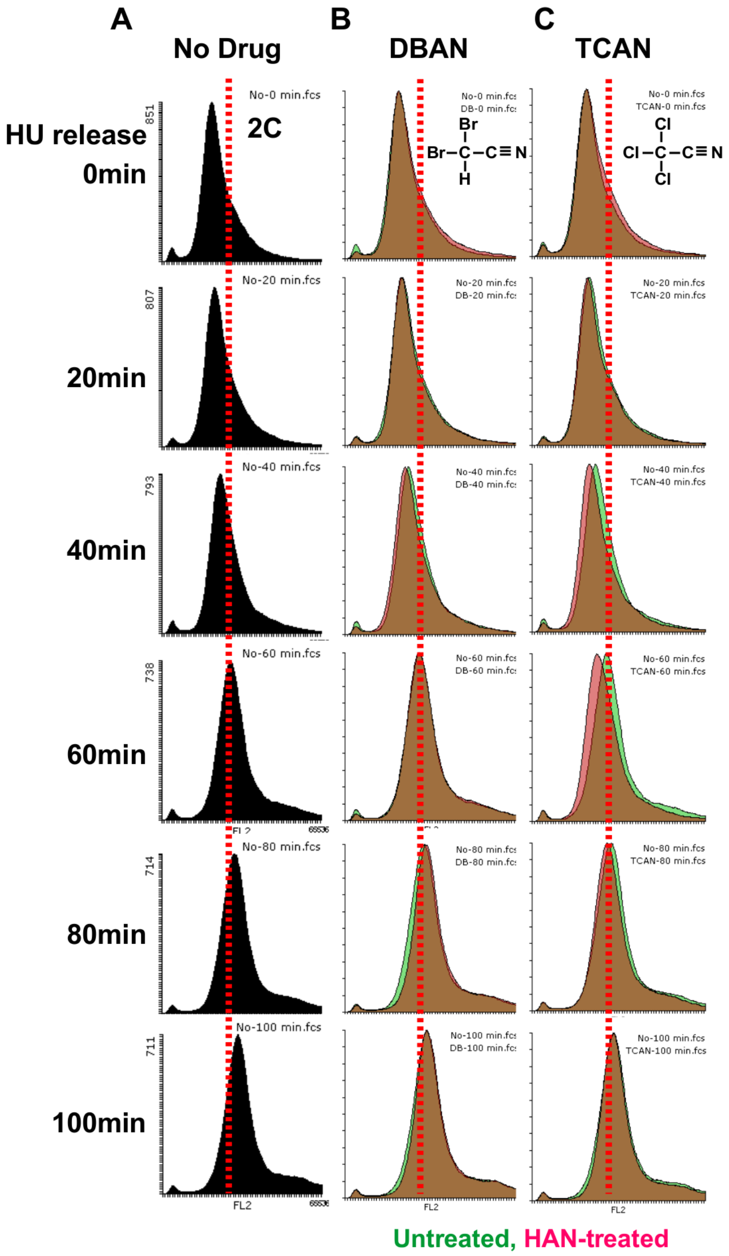

Supplementary Figure S2.

Wild type cells (*ade6-M210 leu1–32 ura4-D18*) were synchronised in early S for 3.5h with 15mM HU at 30°C and released into pre-warmed rich medium without a drug **(A)**, with or without 10µM DBAN **(B)**, and with or without 10µM TCAN **(B)**. The DNA content of untreated cells (green) and cells in the presence of the HAN (red) is shown. The dotted line indicates cells with a 2C (G2) DNA content. The brown colour indicates that both histograms overlap.

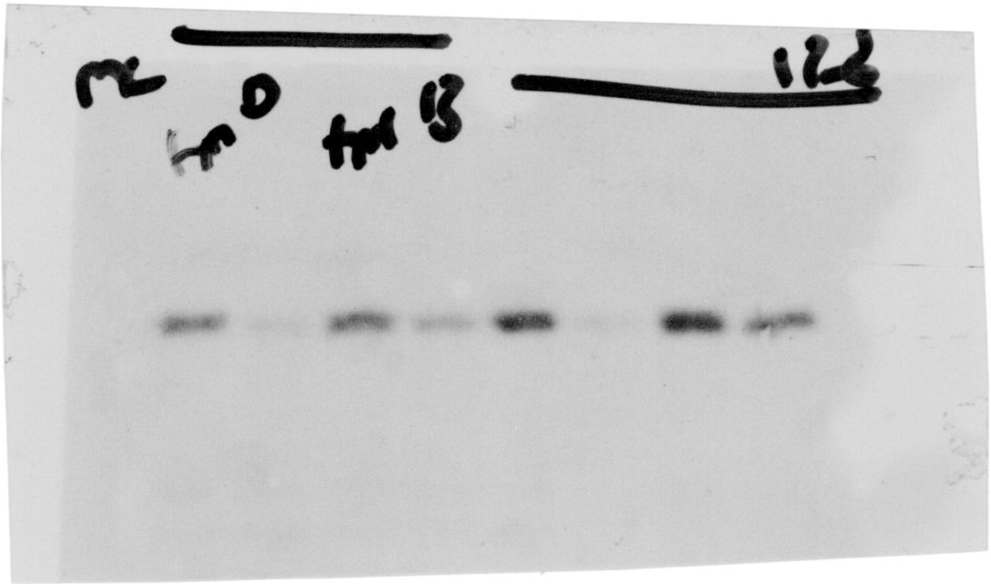

Supplementary Figure S3.

Full image of the H2AX-P-S129 Western Blot (Signal at 16kDa) (ABCAM 17576). Cds1 samples left, Chk1 samples right. Panels shown in Fig. 5A, B.

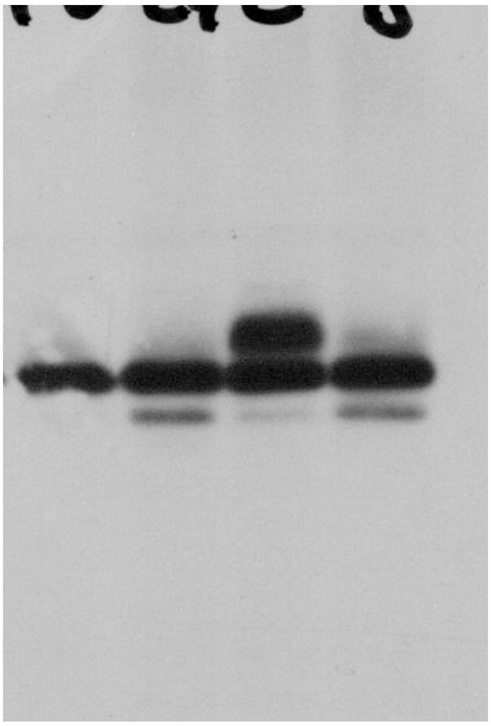

Supplementary Figure S4.

Full image of the Chk1-HA_3 Western Blot (Signal at 60kDa) (Covance AB). Panel shown in Fig. 5B.

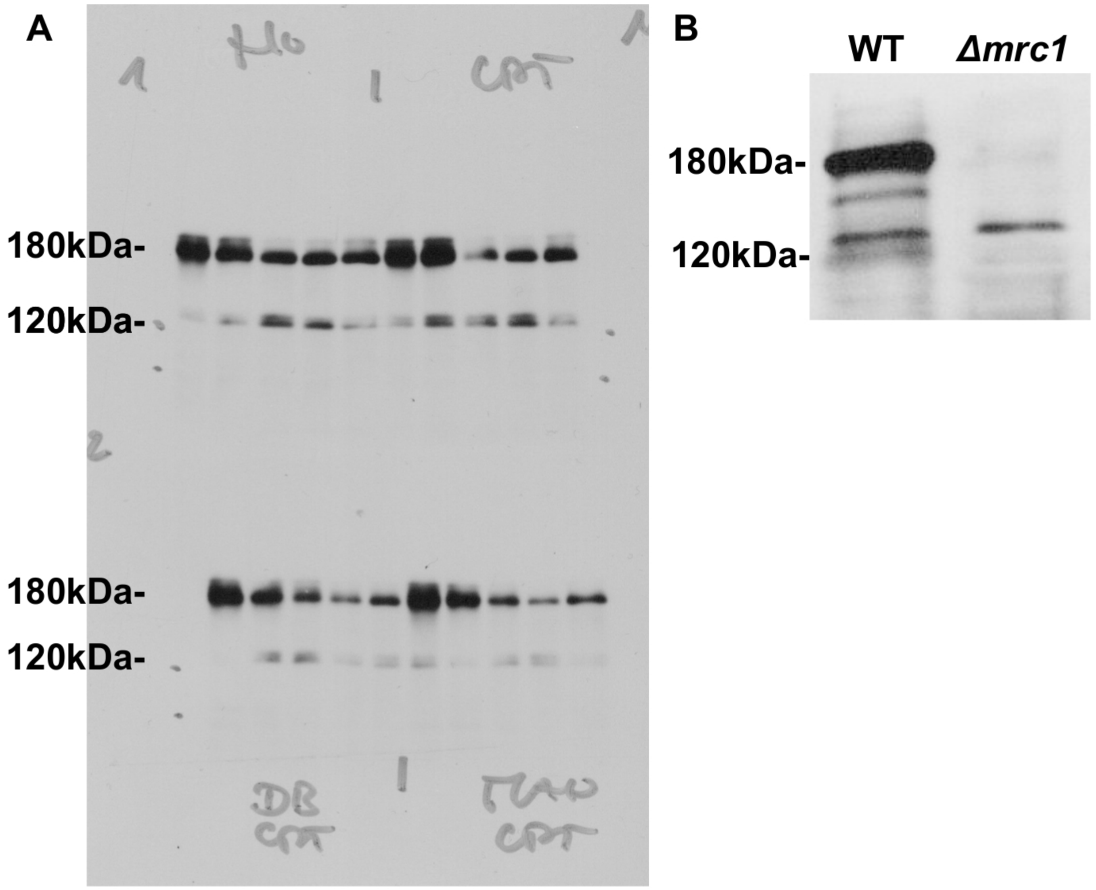

Supplementary Figure S5.

(A) Full image of the Mrc1 Western Blot (ABCAM 188269). The specific band has an apparent molecular weight of 180kDa. There is an unspecific band at 120kDa. **(B)** Western blot of untreated wild type and *mrc1* deletion (*Δmrc1*) strains. Panels shown in Fig. 5E, F.

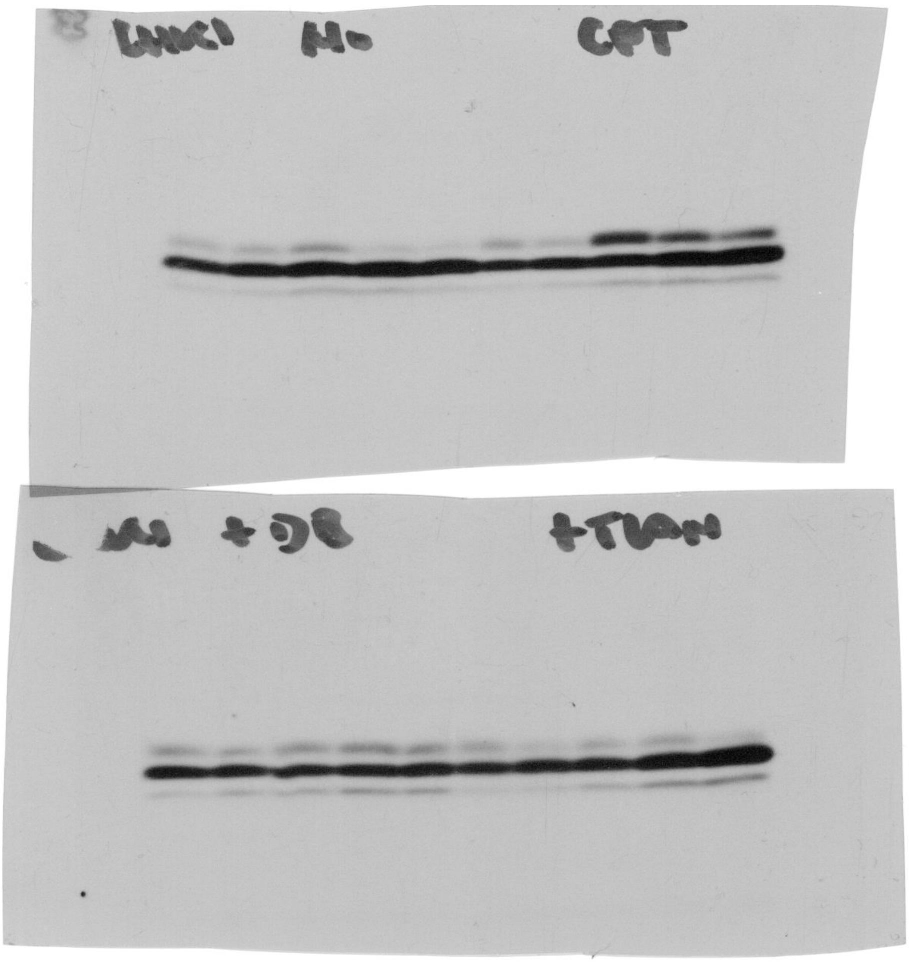

Supplementary Figure S6.

Full image of the Chk1 Western Blot (Signal at 60kDa) (Covance AB). Panels shown in Fig. 5E, F.

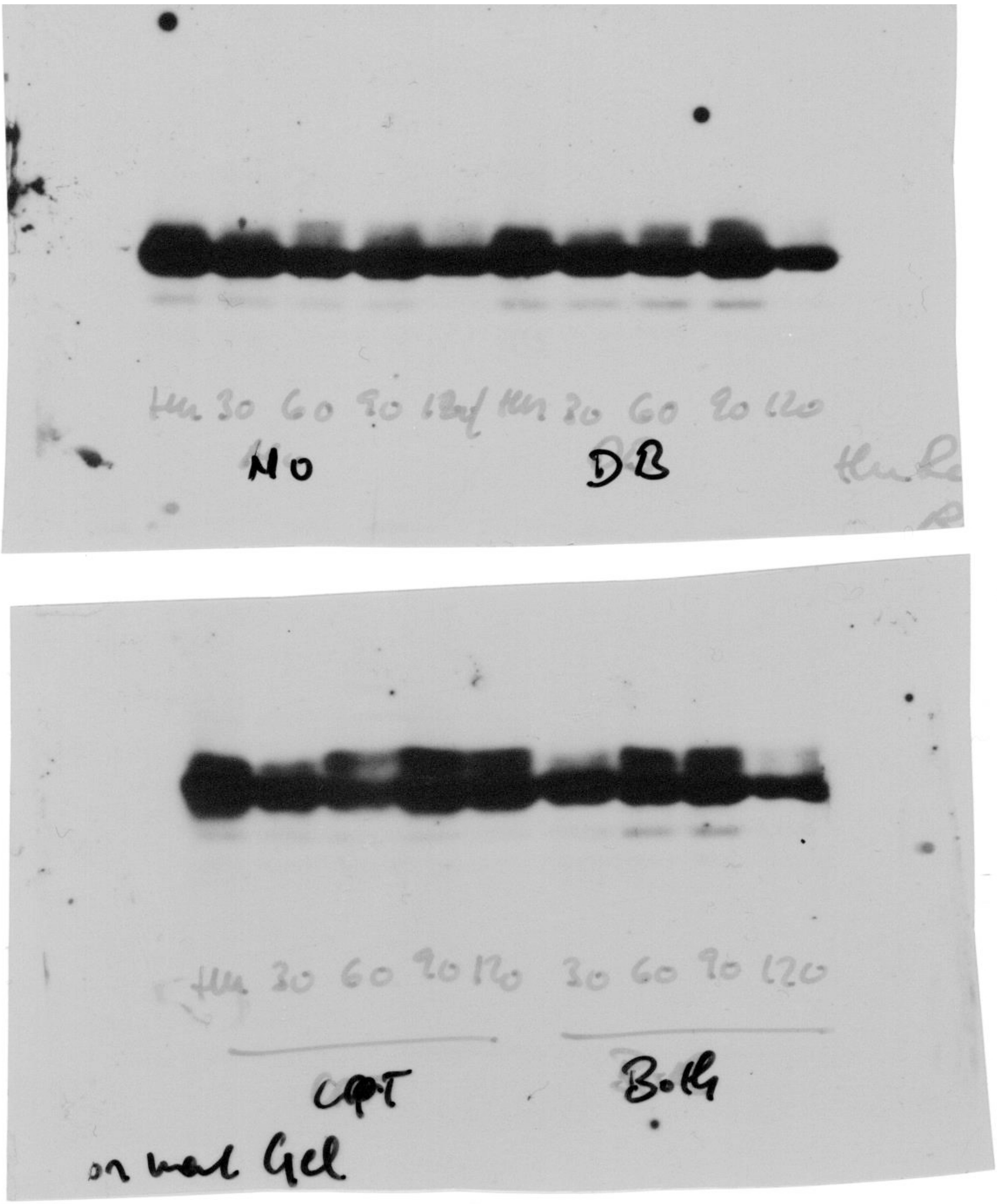

Supplementary Figure S7.

Full image of the Rad9 Western Blot (Signal at 50kDa) (Covance AB). Panels shown in Fig. 5G.

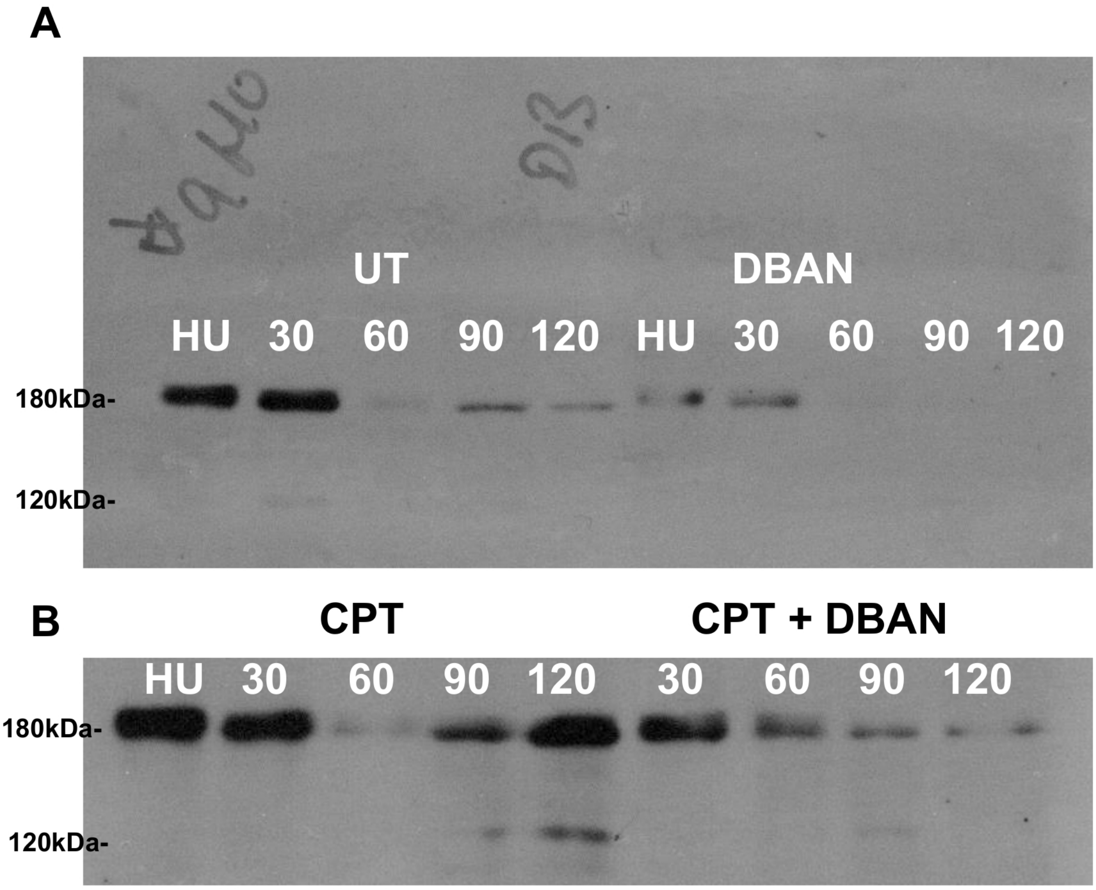

Supplementary Figure S8.

The extracts shown in Fig. S7 were re-probed with the anti-Mrc1 antibody (ABCAM 188269). The films darkened in the laboratory journal. UT = untreated. HU = sample taken after 3.5h in 15mM HU, not shown in Fig. 5G. Panels are shown in Fig. 5G. The Mrc1 specific band = 180kDa. Unspecific band = 120kDa (see Fig. S5).
